# Reconfigurable logic via gate controlled domain wall trajectory in magnetic network structure

**DOI:** 10.1038/srep20130

**Published:** 2016-02-03

**Authors:** C. Murapaka, P. Sethi, S. Goolaup, W. S. Lew

**Affiliations:** 1 School of Physical & Mathematical Sciences, Nanyang Technological University, 21 Nanyang Link, Singapore 637371.

## Abstract

An all-magnetic logic scheme has the advantages of being non-volatile and energy efficient over the conventional transistor based logic devices. In this work, we present a reconfigurable magnetic logic device which is capable of performing all basic logic operations in a single device. The device exploits the deterministic trajectory of domain wall (DW) in ferromagnetic asymmetric branch structure for obtaining different output combinations. The programmability of the device is achieved by using a current-controlled magnetic gate, which generates a local Oersted field. The field generated at the magnetic gate influences the trajectory of the DW within the structure by exploiting its inherent transverse charge distribution. DW transformation from vortex to transverse configuration close to the output branch plays a pivotal role in governing the DW chirality and hence the output. By simply switching the current direction through the magnetic gate, two universal logic gate functionalities can be obtained in this device. Using magnetic force microscopy imaging and magnetoresistance measurements, all basic logic functionalities are demonstrated.

Spintronics, which combines the spin degree of freedom with the charge transport characteristics of the electrons, has the potential to change the basic principles of logic operations in microelectronics devices^1^,^2^. By exploiting the binary state of electron spin in nanomagnets for logic operation, an all-magnetic logic computing architecture is possible, which has the advantages of non-volatility, zero quiescent power, high scalability and high speed. The manipulation of the magnetization direction of nanomagnets, via magnetic field^3^,^4^,^5^,^6^,^7^,^8^ or spin transfer torque effect^9^, has spawned numerous techniques for magnetic logic^3^,^4^,^5^,^6^,^7^,^8^,^9^,^10^,^11^,^12^. In these logic schemes, different physical designs are needed for full logic functionalities. However, a logic which can be reconfigurable at run-time shall simplify the device architecture by eliminating interconnects and can potentially increase the computing speed. The reconfigurability makes the device attractive as a single device can then be programmed for many applications^13^. Though conceptual proposals^14^,^15^ of programmable spin logic exist, experimental demonstration of an all-magnetic reconfigurable logic is still lacking. Semiconductor based reconfigurable logic schemes are demonstrated combining the magnetism and semiconductor technologies^16^,^17^. The logic operations in the device are performed by controlling the motion of charge carriers in p-n junction diodes by the application of a magnetic field. Simple circuit combinations of diode structures are used to perform various logic operations.

Here, we present a prototype of a purely magnetic reconfigurable logic device. The device is capable of performing all basic logic operations on a single magnetic structure. The logic functionality in the device is attained by controlling the motion of a domain wall (DW) in the ferromagnetic asymmetric network structure. The programmability of the device is achieved by using a magnetic gate. The magnetic gate influences the DW trajectory in the network structure by exploiting its inherent transverse charge distribution. By switching the polarity of the magnetic gate, two universal logic functionalities are obtained in this structure.

## Results

**Figure 1:**
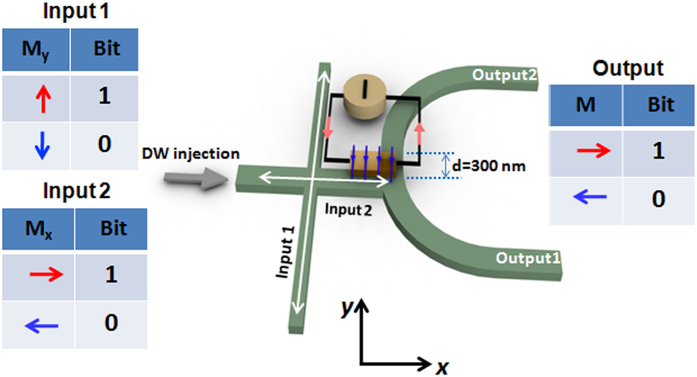
Reconfigurable domain wall logic. Schematic depiction of an all-magnetic reconfigurable domain wall logic. The magnetization orientations along the vertical and horizontal nanowires are considered as input 1 and input 2, respectively. The output is read at the magnetization direction of the upper half-ring and lower half-ring. The half-ring is asymmetric with respect to the horizontal nanowire as it is displaced towards +*y*-direction. A non-magnetic metallic strip line is placed at the bifurcation which generates Oersted field when current is flown through it. The strip line acts as a magnetic gate to select between different logic operations. Also shown are the binary representations of the input and output according to the magnetisation direction of the ferromagnetic elements.

Shown in Fig. 1 is schematic of the all-magnetic reconfigurable domain wall logic device. The device comprises of two nanowires that are orthogonal to each other and a half ring. The magnetization orientations of the two nanowires represent the input bits: the vertical nanowire corresponds to ‘Input 1’, and the horizontal nanowire acts as ‘Input 2’. The magnetization orientations of the individual branches of the half ring represent the logic output operation, as shown in the inset of Fig. 1. In this device, clocking is achieved *via* a global in-plane magnetic field. The device is clocked by an alternating magnetic field pulse. For each clock cycle, the initial output state is pre-set to the logical state of input 2. In this device, logic “1” is defined as the magnetization pointing in the positive direction along the coordinate axis (+*x* or +*y*) and logic “0” as the magnetization pointing in the negative direction (−*x* or −*y*). The output can be programmed by flowing current through a metallic strip which is patterned on top of the structure at the intersection between the horizontal nanowire and the half ring. The metallic strip functions as a magnetic gate due to the Oersted field generated around it in the presence of current. By controlling the polarity of the magnetic gate, the device is able to perform multiple logic operations, *i.e*. NAND, NOR, AND, OR, NOT, and COPY.

**Figure 2:**
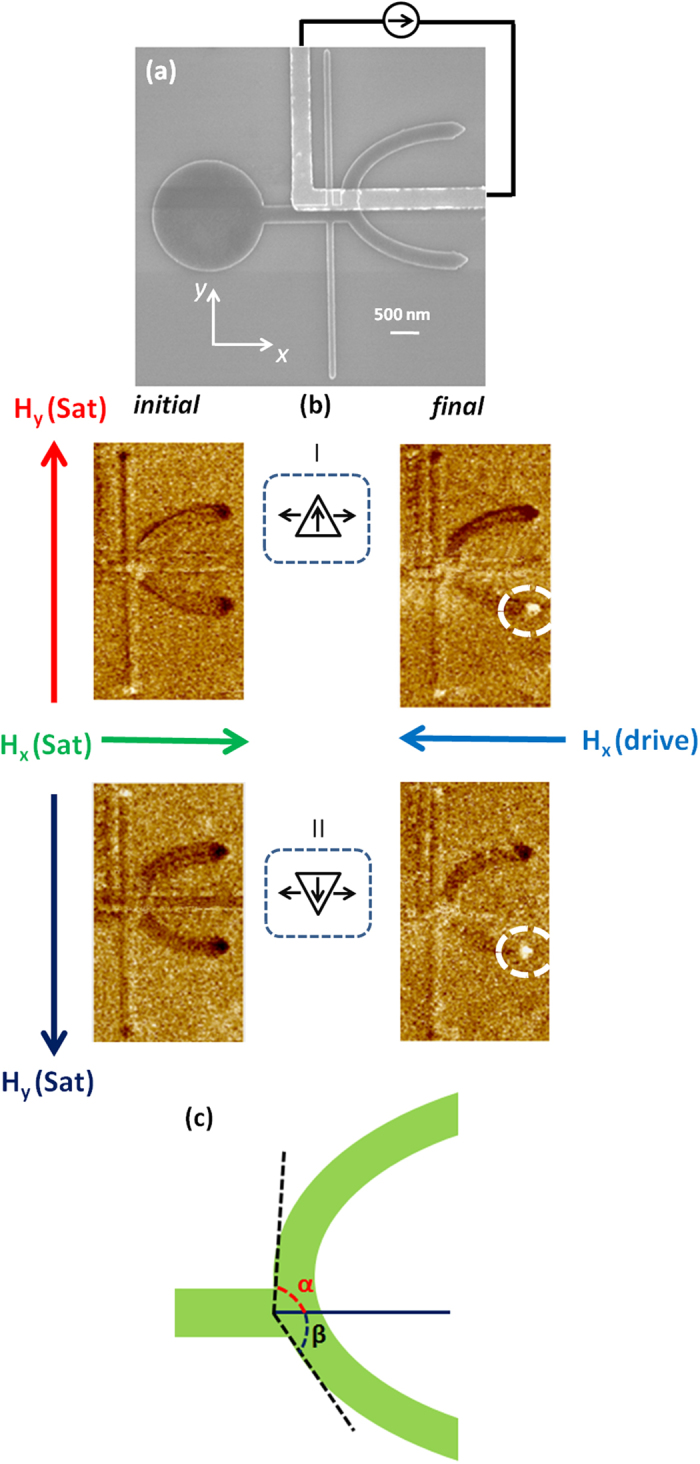
Domain wall trajectory in asymmetric half-ring. (**a**) Scanning electron microscopy (SEM) image of the fabricated device with schematic of the circuit for current injection. (**b**-I) Magnetic force microscopy (MFM) images of the initial and final configurations of the device when a tail-to-tail DW with “Up” chirality (TT-U) is injected and driven. The TT-U is moved towards the lower half-ring and switches the magnetization direction (b-II) MFM images of the initial and final configurations of the device when a tail-to-tail DW with “Down” chirality (TT-D) is injected and driven. TT-D also moves along the lower half-ring and switches its magnetization direction (**c**) Schematic to show the relative angles of the branches along the upper and lower half-rings deviated from the horizontal nanowire when the half-ring is displaced towards +*y*-direction.

Figure 2(a) shows the scanning electron microscopy (SEM) image of the device structure. The ferromagnetic nanostructure is patterned from Ta/Ni_81_Fe_19_/Ta film. The width of the horizontal nanowire is 300 nm. At these dimensions, vortex DWs are stable configurations^18^,^19^ within the nanowire and shape anisotropy constrains the magnetization to follow the long axis of the wire. A circular pad is used for DW injection into the horizontal nanowire. The vertical nanowire (width of 120 nm and length of 6 μm) is placed 100 nm away from the bifurcation to transform the DW from vortex to transverse configuration (discussed in detail later). The half ring is displaced along +*y* direction with an offset of 300 nm to introduce an asymmetry at the bifurcation. The metallic strip (magnetic gate) of Cr(3 nm)/Au(30 nm) is patterned so as to overlap the upper edge of the horizontal nanowire.

We first discuss the device operation without switching on the magnetic gate *i.e*. zero current through metallic strip. Figure 2(b) shows magnetic force microscopy (MFM) images of the device structure. In the initial condition of the state I, the magnetisation orientation of the vertical nanowire is saturated along the +*y* direction, while that of the horizontal nanowire is saturated along the +*x* direction. This is confirmed by the dark and bright contrasts at the edges of the vertical nanowires and half ring in the MFM image where in our convention, the magnetization is always aligned from bright to dark contrast. After applying a linear magnetic field of 100 G along the -*x* direction, a tail-to-tail DW with up chirality is injected and driven which subsequently switched the LHR magnetization. This is evidenced by a change in magnetic contrast of LHR from the dark to bright in the MFM image of the final condition of state I. In state II, where the magnetization orientation of the vertical nanowire is reversed (−*y* direction), a tail-to-tail DW with down chirality is injected and similar switching behaviour at the LHR was obtained.

The deterministic propagation of the DW irrespective of its chirality into LHR in both states can be understood from potential barrier experienced by the DW at the bifurcation. With zero offset at the bifurcation *i.e*. in symmetric structure, the DW experiences similar potential barriers along the UHR and LHR. The motion of the DW is determined by the arrangement of the topological edge defects making it chirality dependent^20^,^21^,^22^. However, when we introduce an offset at the bifurcation, the potential barriers experienced by the DW are different in both directions. As shown in Fig. 2(c), the branch along the UHR deviates with a higher angle (α) at the bifurcation as compared to the branch along the LHR (β) in the asymmetric structure. It shows that DW experiences relatively higher potential barrier along the UHR as compared to the LHR. The steep barrier along UHR constrains the DW motion along the LHR irrespective of its chirality. Micromagnetic simulations^23^ were performed to determine the minimum offset required to constrain the DW motion along one of the branches. It was observed that when the offset is below 50 nm, the DW still follows chirality dependent selective switching above which the DW trajectory is constrained and independent of the chirality.

**Figure 3:**
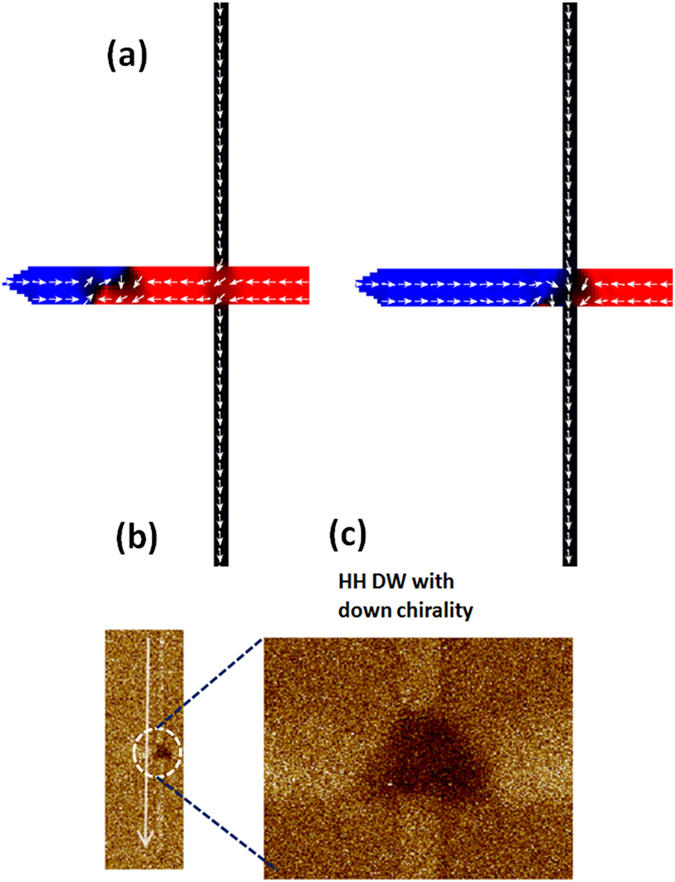
Domain wall transformation at the vertical nanowire. (**a**) Magnetization configuration of a vortex DW relaxed in the nanostructure (**b**) Magnetization configuration of the transverse DW transformed from the vortex configuration which is pinned under the vertical nanowire when driven by magnetic field (**c**) MFM image of the head-to-head DW with down chirality that is pinned under the vertical nanowire along with the zoom in image to show the triangular shape of the transverse DW configuration.

As the DW motion is constrained along one branch due to asymmetry, we need an additional control to perform the logic operation. We have explored the transverse magnetic charge distribution of a transverse DW to impose the additional control. The vertical nanowire placed at the bifurcation helps in the DW transformation from the vortex to transverse configuration. The DW transformation at the intersection of vertical and horizontal nanowires was also studied by micromagnetic simulations and MFM imaging. Figure 3 shows the DW transformation from the head-to-head clockwise vortex DW to transverse DW (TDW) with a down chirality. Our results reveal that vortex DW irrespective of its initial chirality (clock wise or anti-clockwise) turns into transverse DW configuration and the chirality of the transverse DW is defined by the magnetization orientation (±*y*) of the transverse nanowire^24^. The TDW assumes a characteristic triangular shape which gives rise to a transversely varying magnetic charge concentration. The base of the triangular spin structure always has higher charge component^25^. The charge distribution of transverse DW depends on the orientation of the magnetic moment at the wall. Head-to-head TDW (HH-DW) possesses a positive magnetic charge while tail-to-tail TDW (TT-DW) possesses a negative magnetic charge, respectively^25^.

**Figure 4:**
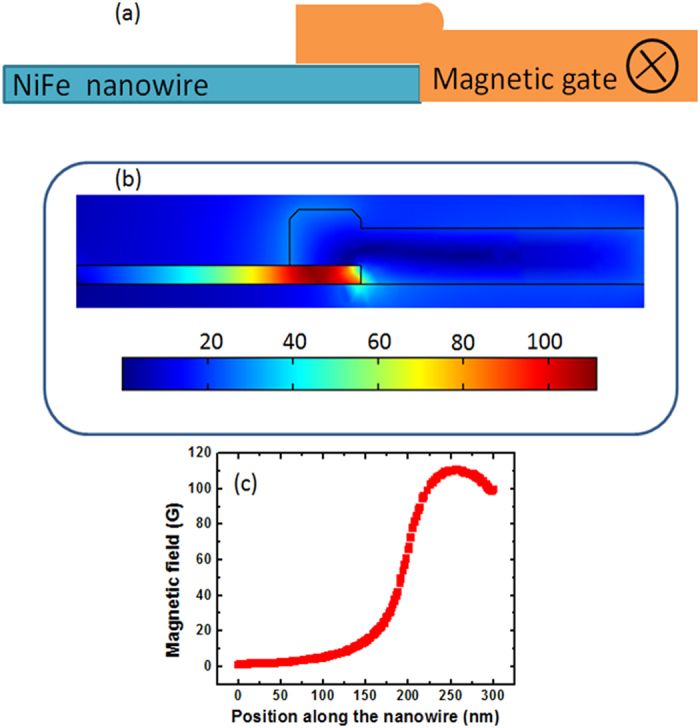
Simulated Oersted field due to magnetic gate. (**a**) Schematic to show the cross-sectional view of the metallic strip line overlapped with the NiFe nanowire. The current flows perpendicular to the plane of paper (**b**) Simulated Oersted magnetic field distribution along cross-section of the NiFe due to the current flowing along the Gate (**c**) Magnetic field strength as a function of position along the NiFe nanowire. The magnetic field is higher beneath the magnetic gate and it drops as we move away from the magnetic gate.

The magnetic gate patterned at the upper edge of the horizontal nanowire influences the DW motion into the half ring depending on the polarity and the charge distribution of TDW. The Oersted field distribution around the magnetic gate is estimated by COMSOL simulation^26^. Shown in Fig. 4(a) is simulated model in which the magnetic gate is overlapped with only 25% of the nanowire width. The transverse magnetic field distribution in the NiFe due to the current is shown in Fig. 4(b). It shows that the top edge of the NiFe which is directly beneath the Au pad experiences a higher magnetic field compared to the bottom edge of the NiFe which is away from the Au pad. The strength of magnetic field generated by current is plotted as a function of position along the NiFe in Fig. 4(c). The field strength is estimated to be ~110 Gauss beneath the Au pad while it is ~10 Gauss at the bottom edge. The local Oersted field interacts with the magnetic charge of the TDW and influences the motion of TDW having higher charge concentration along the upper edge. This dual control of selective motion of the TDW allows the device to perform programmable logic operation within a single structure. By simply changing the direction of the local Oersted field, two universal logic gate operations can be obtained.

### NAND and AND Boolean logic operations

**Figure 5:**
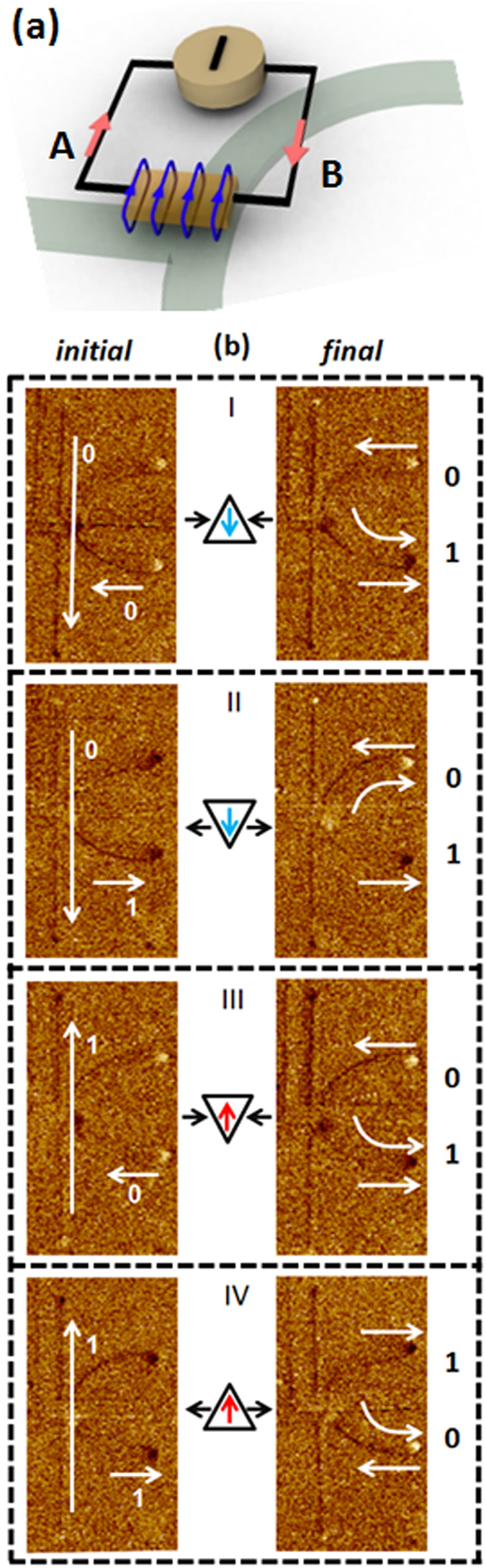
Logical NAND, AND, NOT & COPY operations. (**a**) Schematic of the device with orientation of the Oersted field and the current. The current is flown from B to A through the metallic strip. The red arrow represents the current direction and the blue lines represent the direction of current induced Oersted field. The Oersted field generated at the magnetic gate attracts the TT DW and repels the HH DW in this configuration (**b**) MFM images of the initial and final configurations of the structure for four different combinations of the magnetisation directions of chirality selector (***y***) and the nanowire (***x***). For (“0” & “1”) combination, a TT-D is injected which is attracted towards the upper half-ring as the Oersted field can overcome the potential barrier generated by the asymmetry at the bifurcation. In the all other three cases, the DWs are driven towards the lower half-ring as the asymmetry dominates over the Oersted field at the bifurcation.

Here we discuss in detail the experimental verification of the NAND and AND logic gate operations. A current of 6 mA is passed from B to A through the metallic strip as shown in the schematic of Fig. 5(a). The Oersted field generated curls around the metallic strip attracts negative magnetic charges and repels positive magnetic charges.

#### Inputs “0” and “0”

The initial configuration with both input bits in logical “0” state is captured by using MFM as shown in Fig. 5(b)-I. The magnetisation orientations of Input 1 and Input 2 are both pointing along the negative direction (−*y* and −*x*, respectively). Both states of the UHR and LHR are in logical bit “0”. Following the application of a linear magnetic field of 100 G along +*x* direction, a HH-DW is injected into the nanowire. The transverse component of the HH-DW points in the –*y* direction (down chirality) as dictated by Input 1 (vertical nanowire). After the application of a linear magnetic field, the MFM image shows a change in magnetic contrast of the LHR from bright to dark. The effect of the Oersted field from the magnetic gate on the HH-DW with down chirality is negligible as the higher charge concentration of the DW is at the lower edge of the nanowire. Hence the DW is influenced by the asymmetry resulting in the switching of the LHR magnetisation from –*x* to +*x* direction. Hence the output state at the LHR is logical bit “1”, while that for the UHR is logical bit “0”.

#### Inputs “0” and “1”

In this particular logic combination, the magnetisation orientation of the horizontal nanowire (Input 2) is aligned along the +*x* direction. This configuration for Input 2 presets the outputs of the device (UHR and LHR) to logical “1”. Following the application of a linear magnetic field of 100 G along the –*x* direction, a TT-DW propagates through the horizontal nanowire (Input 2). MFM imaging after the field application shows a change in the magnetic contrast at the UHR from dark to bright, as seen in Fig. 5(b)-II. The transverse component of the injected TT-DW points to the −*y* direction (down chirality) as dictated by the vertical nanowire (Input 1) magnetic configuration. For a TT-DW with down chirality, the higher charge concentration is at the upper edge of the horizontal nanowire. In this case, the attraction from the Oersted field overcomes the potential barrier created by the asymmetry at the bifurcation. The Oersted field from the magnetic gate attracts and guides the TT-DW into the UHR. Consequently, the UHR magnetisation orientation is switched, representing a change from logical bit “1” to “0”. The magnetisation of the LHR remains unchanged, *i.e*. at logical bit “1”. Without the Oersted field from the magnetic gate, the TT-DW would move into the LHR as dictated by the asymmetry of the structure. For our design, the minimum current density required to generate the Oersted field to overcome the potential barrier created along the UHR was ~5 × 10^11 ^A/m^2^.

#### Inputs “1” and “0”

In this configuration, the magnetisation orientation of Input 1 is aligned along +*y* direction (“1”) and the magnetisation orientation of Input 2 is aligned along the −*x* direction (“0”). The MFM image of the final state, following the application of 100 G driving magnetic field along +*x* direction, is shown in Fig. 5(b)-III. The magnetic contrast change at the LHR indicates the switching of magnetisation orientation of the LHR. A HH-DW with an up chirality is injected at the bifurcation as the magnetisation orientation in Input 1 is aligned along +*y* direction. Similar to the case of “0” and “0”, the HH-DW is influenced by both the local Oersted field and asymmetry. As such, the HH-DW moves into the LHR resulting in the reversal of the magnetisation orientation to +*x* direction, which corresponds to an output bit of “1”. The logical state of the UHR remains unchanged as logical bit “0”.

#### Inputs “1” and “1”

 When the magnetisation orientations of the vertical and horizontal nanowires are aligned along the +*y* and +*x* directions, respectively, both logical input bits (Input 1 and Input 2) are “1”. Under the external magnetic field, a TT-DW with an up chirality is injected into the structure. For this DW configuration, the higher charge concentration is along the lower edge of the nanowire. MFM image of the structure after 100 G field application along –*x* direction shows that the magnetisation orientation of LHR has switched as seen in Fig. 5(b)-IV. The effect of the Oersted field from the magnetic gate on the TT-DW with up chirality is negligible as the higher charge concentration of the DW is at the lower edge of the nanowire. The effect of asymmetry overpowers the attraction from local Oersted field at the bifurcation. This leads to the TT-DW motion into the LHR which switches the magnetisation orientation corresponding to an output bit of “0” while that for the UHR remains unchanged as a logic bit of “1”.

The logical output at the UHR and LHR for all the different input combinations are summarized in the truth table in Table 1. For all the logic operations, the resulting magnetic configuration of the UHR and LHR are complementary. For current flowing from B to A through the metallic strip, the outputs at the LHR reveal a NAND logic gate operation, while an AND logic gate operation is obtained at the UHR. The striking feature of this device is that in a single clock cycle, two complementary logic operations can be performed simultaneously.

### NOT and COPY Boolean logic operations

Single Boolean logic bit operation was achieved by fixing Input 1 (vertical nanowire) to logical bit “1” (magnetisation orientation along +*y* direction) and flowing current from B to A through the metallic strip. When the structure was saturated along the −*x* direction, which corresponds to the bit “0” in input 2, the DW propagation leads to the switching of LHR, resulting in a logical “1” output as shown in Fig. 5(b)-III. For logical input bit “1”, the output at LHR reveals a logical bit “0” as presented in Fig. 5(b)-IV. The output bit at the UHR always follows the input bit. The truth table formed in Table 2 shows the structure operates as logical NOT gate when the output is read at the LHR while a COPY operation is obtained at the UHR in this configuration.

### NOR and OR Boolean logic operations

**Figure 6:**
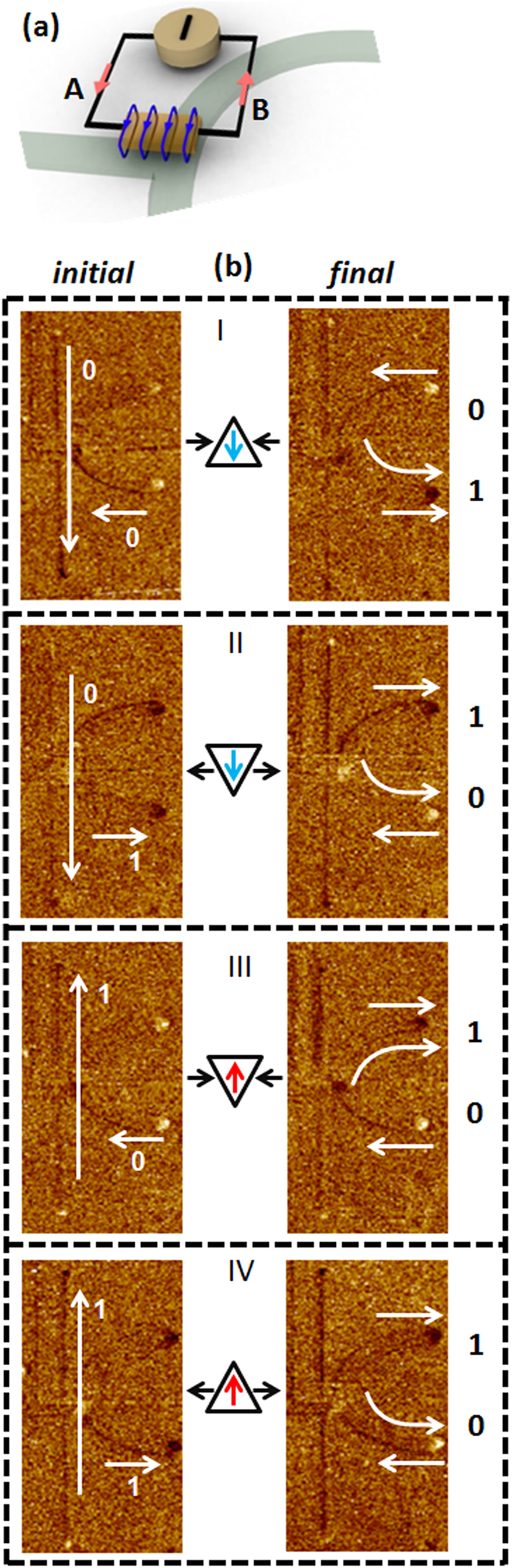
Logical NOR & OR operations. (**a**) Schematic of the device with orientation of the Oersted field when the current is flowing from A to B through the metallic strip. The Oersted field attracts the HH DW and repels the TT DW in this configuration (**b**) MFM images of the initial and final configurations of the structure for four different combinations of the magnetisation directions of chirality selector (***y***) and the nanowire (***x***). Here, for (“1” & “0”) HH-U is attracted towards the upper half-ring as Oersted field overcomes the potential barrier. In th rest of the input combinations, the DW is driven towards the lower half-ring as dictated by asymmetry at the bifurcation.

By reversing the direction of the current flow through the metallic strip *i.e*. from A to B, the polarity of the local Oersted field can be switched, as shown in Fig. 6(a). The local Oersted field now attracts the DW with a positive charge but repels DW with a negative charge. From the NAND operation discussion, the magnetic gate has no effect on the DW when the two input bits are the same, *i.e*. input 1 and input 2 are “0”(“1”) and “0”(“1”), respectively as the charge is concentrated at the lower edge of the nanowire. Therefore the output remains the same for these configurations irrespective of the polarity of the Oersted field as seen in Fig. 6(b)-1&IV. Here we only discuss the other two input combinations. As shown in Fig. 6(b)-II, when Input 1 is logic bit “0” and Input 2 is logic bit “1”, TT-DW with a down chirality is injected at the bifurcation with the application of magnetic field. As the resulting DW has a higher charge concentration (negative) along the upper edge of the nanowire, it is repelled by the field from the magnetic gate. The DW is pushed into the LHR and switches the magnetisation orientation of the LHR leading to a logical output bit “0”. This is clearly observed by the magnetic contrast change from dark to bright at the LHR, as shown in Fig. 6(b)-II.

When Input 1 is logical bit “1” and Input 2 is logical bit “0”, respectively, a HH-DW with an up chirality is formed at the bifurcation. This DW is characterized by a higher (positive) charge concentration along the upper edge of the nanowire. The HH-DW is attracted by the Oersted field from the magnetic gate, and is guided into the UHR. The magnetic contrast at the UHR switches from bright to dark after the logic operation, as seen from the MFM image in Fig. 6(b)-III. The results for the logical operation, where the magnetic gate programmed with current flowing from A to B, are summarised in the truth table, Table 3. The logical output at the LHR shows a NOR gate operation. As the UHR is complimentary to the LHR, a logical OR gate operation is obtained at the UHR. The experimental results clearly show that the device can be programmed to perform both universal logic operations (NAND and NOR) by changing the direction of current flow through the metallic strip.

### Verification of logic functionality by magnetoresistance measurements

**Figure 7:**
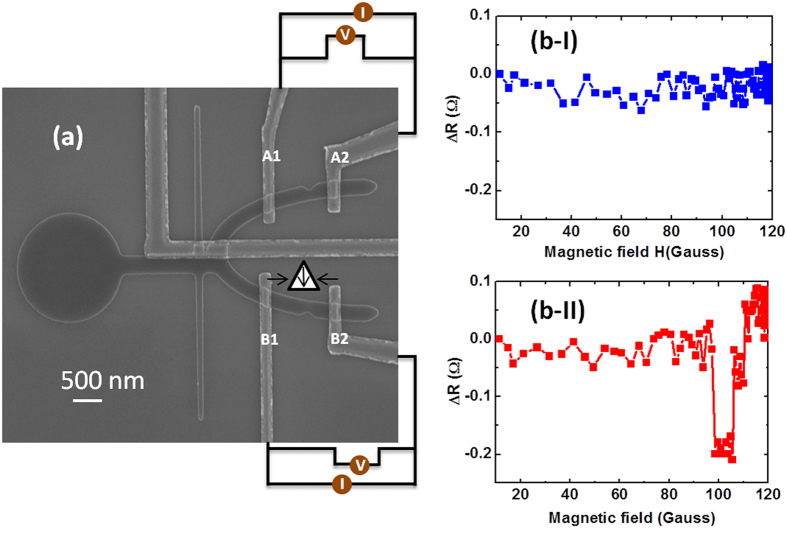
Magnetoresistance measurements. (**a**) Scanning electron microscopy (SEM) image of the device with electrical connections for anisotropic magnetoresistance (AMR) measurements. (b-I&II) AMR signal of the upper and lowers branches during the DW motion through the structure, respectively. A HH-D is injected and driven towards the bifurcation. The DW motion along the lower half-ring can be seen by the change in the magnetoresistance along the lower half-ring. The dip in magnetoresistance shows the pinning and depinning of the DW at the notch structure in the lower half-ring.

We have experimentally demonstrated that multiple logic operations can be performed by a single magnetic structure using MFM imaging technique. The experimental verification of the logical operation can also be carried out using magneto-resistance measurements. As a proof-of-concept demonstration, anisotropic magnetoresistance (AMR) detection measurements^27^,^28^,^29^ have been performed on our sample. As the switching of the individual branches is mediated *via* DW motion, we have directly probed the presence of the DW within the branches. To test the logic operation by electrical means, *in-situ* anisotropic magnetoresistance (AMR) measurements were performed during the logic operation. As the device functionality is mediated via DW motion in the structure, the absence/presence of the DW within the output branch was detected to confirm successful logic operation. Shown in Fig. 7(a) is the SEM image of the device with electrical contacts for AMR measurements. A notch was introduced in each branch to pin the domain wall (DW). It is more accurate and less complex to measure the presence of DW while it is pinned rather than during its motion. The potential difference across each branch (*A1* to *A2* and *B1* to *B2*, as shown in Fig. 7(a) was directly measured *via* dc probes while a constant biasing current of 50 μA was applied.

The *in-situ* AMR plot of the two output branches during the application of the external magnetic field, is shown in Fig. 7(b). ΔR = R(0)-R(H) is the change in the magnetoresistance of the individual branch. The initial configuration of the device was with the input1 set to “0” and input 2 set to “0”, that is, the magnetization of the transverse nanowire (input 1) is saturated in −*y* direction and the longitudinal nanowire (input 2) is saturated along –*x* direction. Application of the magnetic field injects a HH-DW with down chirality in to the longitudinal nanowire. Increase in the magnetic field strength drives the DW towards the bifurcation. AMR measurements at the two branches show that the magnetoresistance between *A1* and *A2* is almost constant as shown in Fig. 7(b)-I. However, ΔR between *B1* and *B2* drops abruptly when the field is increased to 95 Gauss, Fig. 7(b)-II. The change in the magnetoresistance is found to be around 0.2 Ω which is consistent with DW resistance. It reveals that the HH DW with down chirality is constrained to move towards the lower branch but gets pinned at the notch. Further increase in the magnetic field to 110 Gauss shows an increase in the ΔR to initial range which corresponds to the depinning of the DW from the notch. The AMR measurements were repeated 20 times for all combinations of inputs for NAND and NOR logic operations. The results were same as the direct observation by MFM imaging.

## Discussion

We have proposed and demonstrated the prototype of all-magnetic reconfigurable logic device by using MFM imaging and AMR measurements. For on-chip applications, the magnetic signals can be electrically read out using Giant magnetoresistance (GMR) spin valves or Magnetic tunnel junction (MTJ) structures^30^ embedded within the device. The output branch (UHR and LHR) can potentially serve as the free layer of the spin valve or MTJ sensor. The relative orientation between the free layer and the reference layer can be read as a change in the resistance of the sensor. The clocking field used in the current device design can be locally generated by an on-chip inductor in the form of a meander. The magnetization configurations of the device for NAND gate operation during the pulse field are presented in supplementary information. The need for alternate pulse field for switching can be eliminated by exploiting the spin transfer torque (STT) effect on DW^30^,^31^,^32^,^33^,^34^. It is well known that current driven DW is unidirectional irrespective of the type of DW. Hence, our device concept is compatible with STT effect and can be made to be solely current controlled. With the inclusion of a current stripe line for DW injection, and given that the speed of DW in planar nanowires can be 100 m/s or higher^30^,^31^, the operation speed of our logic device can reach to sub-10 ns range. The stability of transverse DW structure is important for our logic operation and the magnetic field of 100 G may lead to DW transformations due to Walker breakdown^35^,^36^. However, the vertical nanowire placed close to bifurcation assists in extending the DW fidelity length to around 350 nm when driven under 100 G field^24^. The distance between the vertical nanowire and the half ring is kept close to 100 nm, well below the DW fidelity length so as to preserve the transverse DW structure during its motion.

To optimize the device footprint, overlay of two structures with a shared magnetic gate may realize a 3-D logic device which will be able to perform four logic functionalities (NAND, NOR, AND and OR) in a single operation. The field orientation at the junction will be in opposite direction for the top and bottom structures. When the top structure acts as a NAND (AND) gate, the bottom structure acts as a NOR (OR) gate at the LHR (UHR). It gives an added advantage of performing two universal logic gate functionalities in a single operation which can pave the way for parallel processing.

## Methods

### Device Fabrication

Magnetic thin films structures of Ta (3 nm)/Ni_81_Fe_19_ (10 nm)/Ta (3 nm) were deposited on pre-cleaned thermally oxidized Si substrates using DC magnetron sputtering techniques. The base pressure of the sputtering deposition system was better than 2 × 10^−8^ Torr. The bottom and top Ta layers act as seed and capping layers, respectively. The magnetic thin film structures were patterned using electron beam lithography and Ar ion-beam etching techniques. Metallic strip at the bifurcation along with the electrical contacts were patterned in the second step of electron beam lithography. A bilayer thin film stack of Cr(3 nm)/Au(30 nm) contact lines was deposited by using electron beam evaporation at a pressure of better than 10^−6 ^Torr. The Cr under layer helps to improve the adhesion of Au on the device. Lift-off of the metallic film was completed in warm acetone to obtain the final device.

### Electrical Measurement Setup

To generate the local Oersted field, a constant current source (Keithley 2400) was used to apply current through the metallic gate. An electromagnet was used as a magnetic field generator for DW injection and driving. The same electromagnet was also used to clock the signal. For AMR measurements two Keithley 2400 constant current sources were used to apply the bias current. The potential difference across each branch (*A1* to *A2* and *B1* to *B2*, as shown in Fig. 7) was directly measured *via* dc probes while a constant biasing current of 50 μA was applied. Two digital voltmeters (Keithley 2000) were used to capture the AMR signal.

### MFM imaging

MFM scanning was carried out using Digital Instruments Dimension 5000 model and commercially available low moment tips. For all our measurements, the tip was magnetized to have a south pole induced at its apex. The measurements were performed in the phase detection mode. The MFM images were taken after applying and removing external magnetic field. To minimize the topography information, a lift scan height of 100 nm was used during the measurements. The bright contrast in the MFM image corresponds to repulsive interaction and net negative magnetic charge according to our convention.

### Micromagnetic Simulation

Object Oriented Micromagnetic Framework (OOMMF) program^23^ was used to perform the micromagnetic simulations. The material parameters for Ni_81_Fe_19_ were chosen: the saturation magnetization, *M*_*S*_ was taken as 8.6 × 10^5 ^Am^−1^, the anisotropy constant, *K* was taken as zero and the exchange stiffness constant *A*, was taken as 1.3 × 10^−11 ^Jm^−1^. The Gilbert damping constant, *α* was chosen as 0.01. A mesh size of 5 × 5 × 5 nm^3^ was employed for all simulations.

## Additional Information

**How to cite this article**: Murapaka, C. *et al*. Reconfigurable logic via gate controlled domain wall trajectory in magnetic network structure. *Sci. Rep*. **6**, 20130; doi: 10.1038/srep20130 (2016).

## Supplementary Material

Supplementary Information

## Figures and Tables

**Table 1:** NAND and AND logic operations.

My	Mx	AND M (UHR) (Output 1)	NAND M (LHR) (Output 2)
↓0	←0	←0	→1
↓0	→1	←0	→1
↑1	←0	←0	→1
↑1	→1	→1	←0

Truth table formed based on the input and output magnetization orientations of the device which shows AND and NAND functionalities at upper half-ring and lower half-ring, respectively.

**Table 2:** NOT and COPY logic operations.

My	Mx	COPY M (UHR) (Output 1)	NOT M (LHR) (Output 2)
↑1	←0	←0	→1
↑1	→1	→1	←0

Truth table formed based on the input and output magnetization orientations of the device for single bit logic operation by keeping the chirality selector fixed along +*y* direction. The results show COPY and NOT functionalities at upper half-ring and lower half-ring, respectively.

**Table 3:** NOR and OR logic operations.

My	Mx	OR M (UHR) (Output 1)	NOR M (LHR) (Output 2)
↓0	←0	←0	→1
↓0	→1	→1	←0
↑1	←0	→1	←0
↑1	→1	→1	←0

Truth table formed based on the input and output magnetization orientations of the device which shows OR and NOR functionalities at upper half-ring and lower half-ring, respectively.
