# Supplementary material for: Reconfigurable logic via gate controlled domain wall trajectory in magnetic network structure
Source: Sci Rep. 2016 Feb 3;6:20130. doi: 10.1038/srep20130 (PMC4738283; doi:10.1038/srep20130)
Supplement: Supplementary Information [file srep20130-s1.pdf]

Supplementary Information

Reconfigurable logic via gate controlled domain wall trajectory in magnetic network structure

C. Murapaka†, P. Sethi†, S. Goolaup and W. S. Lew*

School of Physical & Mathematical Sciences, Nanyang Technological University
21 Nanyang Link, Singapore 637371

NAND gate operation in pulse magnetic field

We have performed micromagnetic simulations of the logic operation in alternating magnetic field. The traces of input and output during the positive and negative magnetic fields are presented for NAND gate operation in Figure S1. As we can see for the input combinations of ("0" & "1") and ("1" & "1"), where a tail-to-tail domain wall (TT DW) is going to be injected in to the horizontal nanowire, the magnetic field in positive direction acts along the saturation of the magnetization and the logic operation is only performed during the negative direction magnetic field. Thus the clock (alternate direction of magnetic fields) does not disturb the output of the logic functionality. However, for the input combinations of ("0" & "0") and ("1" & "0") in which a head-to-head (HH) DW is going to be injected, the logic operation occurs during the positive direction of magnetic field. During the negative direction of magnetic field, a TT DW is injected from the nucleation pad and moves towards the half-ring. When the input bits are ("0" & "0"), a TT DW with down chirality is injected and driven towards the half-ring. Interestingly, the transverse component of the TT DW aligns with the magnetization orientation of the half-ring (counter- clockwise rotation). Thus, the TT DW is annihilated at the bifurcation without disturbing the magnetization of the half-ring (output). When the input is "1" & "0", a TT DW with up chirality is injected into the horizontal nanowire from the nucleation pad during the magnetic field being in the negative direction. When the DW reaches the bifurcation it experiences repulsion [S1] from the magnetization of the half-ring as the transverse component of the DW and the magnetization of the half-ring are aligned in the opposite direction. Due to the repulsion the TT DW is pinned at vertical nanowire which does not move further towards the bifurcation, thus leaving the output same as that during the positive direction magnetic field. These results show that for all four input combinations, the output of the logic operation remains unperturbed during the application of alternate direction pulse magnetic field.

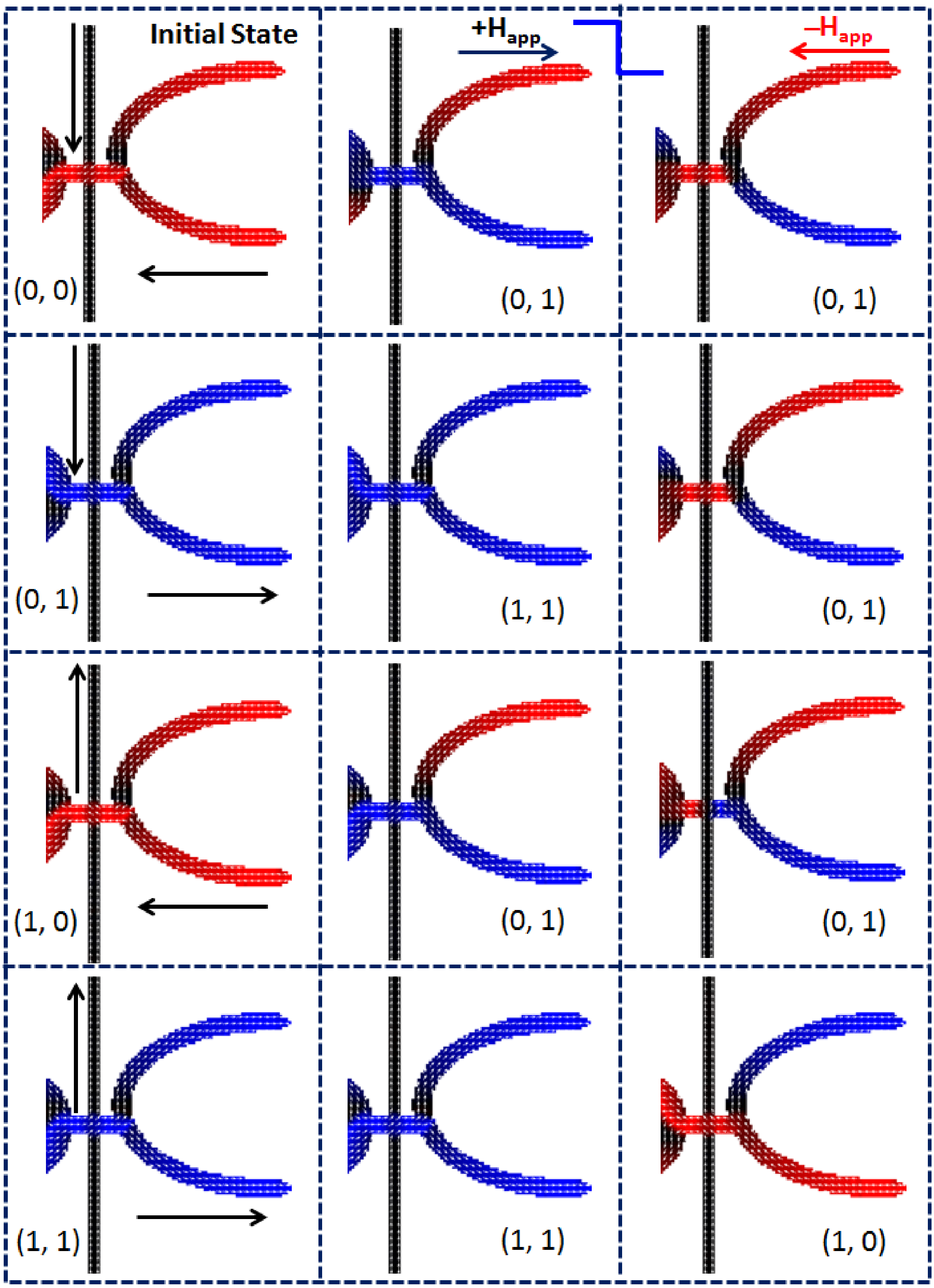

Figure S1. The simulated magnetization configurations of the reconfigurable logic device during NAND gate operation under alternate pulse magnetic field as clock.

S1. Thomas, L. *et al.* Topological repulsion between domain walls in magnetic nanowires leading to the formation of bound states. *Nature Commun.* **3**, 810 (2012).
